# Advances in otolith-related protein research

**DOI:** 10.3389/fnins.2022.956200

**Published:** 2022-07-26

**Authors:** Shouju Huang, Shuxia Qian

**Affiliations:** ^1^The Fourth School of Clinical Medicine, Zhejiang Chinese Medical University, Hangzhou, China; ^2^Department of Neurology, The Second Affiliated Hospital of Jiaxing University, Jiaxing, China

**Keywords:** otolith, development, formation and maintenance, otolith-related protein, benign paroxysmal positional vertigo

## Abstract

Otoliths are biological crystals formed by a layer of calcium carbonate crystal that adhere to the ciliary surface of the utricular and saccular receptors in the vestibule of all vertebrates inner ear, enabling the utricle and saccule to better perceive the changes in linear and gravitational acceleration. However, the molecular etiology of otolith related diseases is still unclear. In this review, we have summarized the recent findings and provided an overview of the proteins that play important roles in otolith formation and maintenance (Otoconin-90, Otolin-1, Otolith Matrix Protein-1, Cochlin, Otogelin, α-Tectorin, β-Tectorin, Otopetrin-1, and Otopetrin-2, PMCA2, etc.), providing new insight for the prevention and management of benign paroxysmal positional vertigo (BPPV) with basis for otolith-related proteins as potential biomarkers of vestibular disease.

## Preface

**FIGURE 1:**
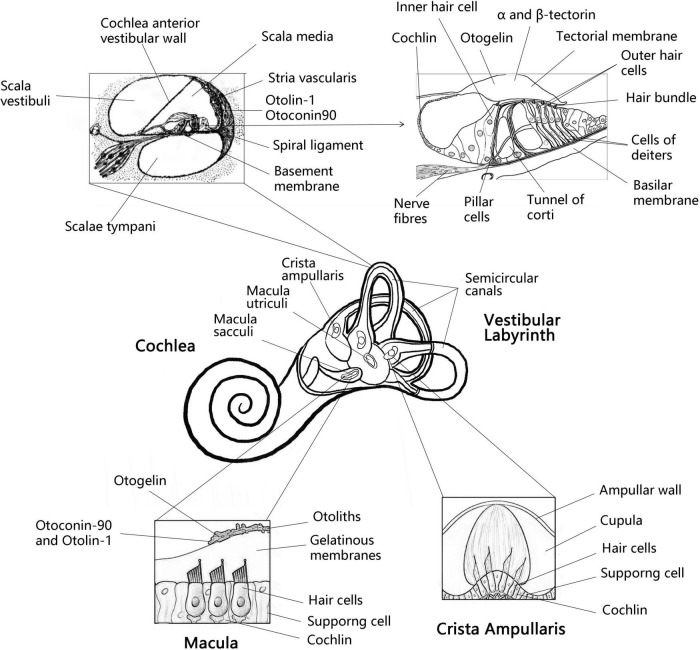
Anatomy the vestibular labyrinth and organ of Corti with labeled structures and locations of inner otolith-related proteins. Anatomy of the vestibular labyrinth with labeled structures and locations of inner ear-exclusive proteins. Adapted from Encyclopaedia Britannica, Inc. 1997, by Jack Dumala.

The inner ear consists of the cochlea and vestibular organs, where the cochlea is responsible for hearing while the vestibular organs perceive the movement of the head, essential for spatial orientation and body balance (Yang et al., 2018). The vestibular organ on each side contains three semicircular canals and two otolith organs (utricle and saccule). The otolithic receptors are distributed on the maculae of the utricle and saccule (Curthoys et al., 2017; Figure 1). Henceforth, the correct formation, fixation and maintenance of the vestibular otolith are necessary to maintain optimal vestibular function and preserve the sense of balance (Boyle and Varelas, 2021). Research has shown that protein fragments must express collagen OC90 (Otoconin-90) and Otolin to induce the structural formation of the otolith crystal (Kao et al., 2017). Therefore, in order to understand the role of otolith in the course of vestibular disease, it is essential to scrutinize the protein and molecular mechanisms underlying the formation and maintenance of otolith (Kao et al., 2017). In this article, combined with the latest findings of zebrafish model, we have reviewed the important otolith related proteins in the formation and maintenance of otolith and their possible contribution in the pathogenesis of vestibular diseases.

## Otolith formation

Unlike bones and teeth which are mainly formed from carbonate hydroxyapatite, otoliths are mainly composed of calcium carbonate crystals (Ross et al., 1976). Otoliths are formed in the late stages of embryonic development and mature shortly after birth, and then remain basically stable (Moreland et al., 2014). Normal otolith formation requires: (1) Correct induction and formation of utricle and saccule; (2) Standardization and differentiation of sensory maculae, sensory cells and Supporting cells; (3) Establishment of correct ionic environment to allow the normal output and processing of matrix proteins and ions; (4) Production and export of otolith matrix protein and membrane; (5) Assembly of the protein core by free floating matrix protein; and (6) Local increase in calcium ion and carbonate concentrations to initiate the formation of crystals on otoliths. The above elements must appear in a specific order and in the correct form at a specific point in time (Hughes et al., 2006). During the growth of otoliths, the adhesion between biomineralized otoliths and cysts and maculae must be maintained and this is achieved through the otolith membrane (Stooke-Vaughan et al., 2015). The key regulator of otolith morphogenesis is considered to be matrix protein. The inhibition of otolith growth may be due to the reduction of calcium carbonate deposition or the lack of matrix protein, or both (Mulry and Parham, 2020). So far, the molecular process of otolith formation and maintenance is still unclear. Eight proteins specific to inner ear are known: Otolin-1, Otoconin 90/95, Prestin, Otoacorin, Otoglin, α-tectorin, β-Tectorin, and Cochlin (Mulry and Parham, 2020; Table 1). There are three main groups of proteins involved in otolith biomineralization: (1) Otolith matrix protein, such as OC90, Otolin-1, OMP-1, Cochlin, etc.; (2) Otolith anchoring protein, the otolith membrane is composed of Otolin and five non-collagenous glycoproteins (Otoglin, Otoglin-like, Otancorin, α-Tectorin, and β-tectorin) and KSPG, which may be primarily associated with otolith anchoring (Tian et al., 2020); (3) Otolith regulatory protein, such as Plasma membrane calcium ATPase isomer 2 (PMCA2), Carbonic anhydrase (CA), Otoptin1 and Otoptin-2, NOx (nitrous oxides), etc. (Tian et al., 2020).

**TABLE 1:** Overview of otolith-related proteins.

Otolith-related proteins or molecules	Location	Role in otolith development and maintenance	Clinical significance
Otoconin-90	Located in the supporting cells of the organ of Corti and the vestibular macula (Lundberg et al., 2015)	The main matrix protein of otoliths, promotes otolith nucleation and growth (Lundberg et al., 2015)	Elevated serum OC90 levels in patients with benign paroxysmal positional vertigo (Bi et al., 2021); Reduced balance in knockout mice (Zhao et al., 2008)
Otolin-1	Located in the supporting cells of the organ of Corti and the vestibular macula (Hołubowicz et al., 2021)	Otolith scaffolding protein, is involved in the formation and correct positioning of the otolith and its correct anchoring to the sensory macula (Weigele et al., 2015)	Elevated serum Ootolin-1 levels in patients with benign paroxysmal positional vertigo (Wu et al., 2020)
Otolith Matrix Protein-1 (OMP-1)	Expressed in the cochlea and vestibular organs (mainly the utricle and saccule) (Weigele et al., 2015)	Maintains normal otolith growth and is directly related to otolith shape (Murayama et al., 2005)	Associated with movement disorders (Weigele et al., 2015)
Cochlin	Expressed in the cochlea and vestibule (mainly in spiral ligament, spiral bone and spiral rim of the inner ear) (Rhyu et al., 2020)	Main otolith constituent, determinant of calcium carbonate crystalline formation (Leventea et al., 2021)	Its overexpression is associated with Ménière’s disease (Calzada et al., 2012); associated with conductive hearing loss and vestibular dysfunction (Leventea et al., 2021; Verdoodt et al., 2021); diagnosis of exolymphatic fistula (Xiong et al., 2020); diagnosis of exolymphatic fistula
Otogelin	Expression in the cochlea and vestibule (Avan et al., 2019)	Anchoring of the otolith (Schraders et al., 2012; Whitfield, 2020); stabilization of the cochlear covering membrane (Avan et al., 2019)	Deficiency leading to otolith detachment and instability of the cochlear covering membrane; OTOG mutation leading to DFNB18B hereditary deafness (Avan et al., 2019)
α-tectorin and β-tectorin	Non-collagenous glycoprotein component of the Cochlear Covering Membrane (TM) (Andrade et al., 2016)	Adherence of TM to the spiral rim and to the stereocilia of the outer hair cells of the cochlea (Andrade et al., 2016) Determinants of otolith formation (Asgharzade et al., 2011)	Deficiency can lead to varying degrees of hearing loss (Asgharzade et al., 2017) β-tectorin deficiency can lead to imbalance (Zou et al., 2006)
Otopetrin1 and Otopetrin-2	Expression in the cochlea and vestibule (Kim et al., 2010; Tu et al., 2018; Khan et al., 2019; Lopez et al., 2019)	Maintains nucleus formation, and growth of otoliths (Kim et al., 2010); a proton-selective channel involved in gravity sensing in the vestibular system	Lack of otopetrin-1 knockout in mice leads to vestibular dysfunction (Khan et al., 2019).
PMCA2	High level of expression in vestibular and outer hair cells (Han et al., 2019)	Maintains otolith formation, and growth (Xu et al., 2021); is a calmodulin-sensitive plasma membrane calcium ATPase.	PMCA2w/a missense mutation causes deafness and balance disorders in humans (Bortolozzi and Mammano, 2018)
Nox (NADPH oxidases)	Expression in the cochlea and vestibule (Kiss et al., 2006; Xu et al., 2021)	The main otolith components and factors necessary for calcium carbonate crystallization (Xu et al., 2021)	The absence of NOX3 enhances hearing recovery after noise trauma (Rousset et al., 2022)
CA (carbonic anhydrase)	Expression in the cochlea and vestibule (Tohse and Mugiya, 2001; Matsumoto et al., 2017; Liu et al., 2022)	Maintaining the right pH to promote otolith development and regulate otolith growth (Tohse et al., 2006; Matsumoto et al., 2017)	CA inhibitors can cause hearing loss, and inhibit calcification of otoliths (Tohse and Mugiya, 2001; Liu et al., 2022)

## Important factors affecting the formation and maintenance of otoliths

### Otolith development and the proteins involved in otolith development

#### Otoconin-90

Otolithic proteins play the role of building matrix in the process of biomineralization and directly control crystal size and polymorph selection (Weigele et al., 2016). During the development of otolith, the organic matrix is formed before the deposition of calcium carbonate. Gene targeting technology has proved that collagen OC90 (otoconin-90) is necessary for the formation of otolith (Zhao et al., 2007). OC90 is the main matrix protein of mammalian calcified otolith: It retains the ability of calcium binding and is extremely acidic, so that calcium or CaCO_3_ can bind. In the absence of OC90, the matrix-bound calcium on the surface of the vestibular maculae is greatly reduced. In both *in vivo* and *in vitro* studies, OC90 has shown to promote the nucleation and growth of CaCO_3_ crystals (Lundberg et al., 2015). Some studies have reported that OC90 deficiency leads to the formation of giant otoliths and that OC90 knockout mouse have reduced balance despite normal hearing (Zhao et al., 2008). Bi et al. (2021) showed that Otoconin-90 levels were significantly higher in subjects with BPPV, that serum otoconin-90 levels were an independent risk factor for benign paroxysmal positional vertigo, and that Otoconin-90 may be a potential biomarker for the development of BPPV.

#### Otolin-1

Otolin-1, a scaffold protein for otoliths, is found in the extracellular matrix of the inner ear and contains a C1q-like structural domain that forms a stable trimer in the presence of calcium ions and is responsible for Ca^2+^ trimerization and binding (Hołubowicz et al., 2017, 2021). OC90 can bind to Otolin-1 and build a protein-rich matrix that serves as a scaffold for subsequent calcium carbonate deposition (Thiessen et al., 2019). Studies have shown that it is particularly involved in the correct positioning of otoliths in fish, so the correct fixation of otoliths on sensory vesicular maculae is necessary. Otolin-1 was not detected in the utricle, so we speculate that it may be a saccule-specific protein linker between the otolith and the otolith membrane (Weigele et al., 2015). Furthermore, Otolin-1 and OC90 can synergistically regulate calcite crystal morphology *in vitro* and contribute to the formation of otolith-like morphology (Moreland et al., 2014). It has been shown that serum Otolin levels reflect otolith status, that Otolin-1 levels are significantly higher in subjects with BPPV, and that Otolin-1 may be a potential biomarker for the pathogenesis of BPPV (Wu et al., 2020). It was also found that both parathyroid hormone and total calcium levels affect the otolin-1 levels of patients with primary hyperfunction of parathyroid, suggesting that calcium dysregulation caused by primary hyperparathyroidism may contribute to otolith catabolism by affecting Otolin-1 levels, and may be associated with inner ear diseases such as BPPV (Mckenna et al., 2020). Assessment of Otolin-1 levels during or shortly after acute BPPV episodes should be considered in future studies (Wu et al., 2020).

#### Otolith matrix protein-1

The cDNA homolog of OMP-1, zomp-1, was found to be necessary for normal otolith growth in the saccule of the adult inner ear by a zebrafish model. OMP-1 is synthesized by most epithelial cells outside the sensory patches (Murayama et al., 2005). In the saccule of *Oreochromis mossambicus*, OMP-1 expression is restricted to the dorsal and ventral reticular areas of the macula sacculi, with the sensory portion lacking expression; In the utricule, OMP-1 was present in the median centripetal meshwork area and lateral centrifugal meshwork area (Weigele et al., 2015). OMP-1 is also the major calcium-binding protein in fish otoliths, and the expression pattern of OMP-1 is directly correlated with the shape of the otolith, and alterations in this expression pattern may lead to impaired locomotion. As fish typically exhibit locomotor behavior after transfer from Earth gravity to microgravity, they can be used as a model system for higher vertebrates (Weigele et al., 2015).

#### Cochlin

Cochlin is expressed in the cochlea and vestibule; normal cochlin expression is evenly distributed in the utricle and the matrix of the ampulla ridge (inner ear protein). Cochlin is involved in clearing bacterial infections in the inner ear, regulating immunity by physically capturing pathogens in the drum stage and recruiting innate immune cells to protect the normal inner ear structure from inflammatory damage (Rhyu et al., 2020). Overexpression of cochlin in the early stage of Meniere’s disease may be one of the causes of intractable Meniere’s disease (Calzada et al., 2012). Verdoodt et al. (2021) found that the loss of Cochlin had a protective effect on hearing threshold after noise exposure in a Cochlin knockout mouse model, but the mutation of cochlin gene encoding cochlin protein can lead to DFNA9, an autosomal dominant non-syndromic sensorineural deafness with vestibular involvement. Cochlin, which is defective in human hearing and vestibular disorders, and DFNA9, is secreted by small specialized areas of vestibular system epithelia whereby cells secrete Cochlin to the ear lumen and base of the basal lamina. Cochlin secreted from the base diffuses along the basal surface of vestibular epithelium, and is apically secreted and integrated into otolith. Cochlin apical secretion defects can lead to abnormal otolith crystallization and behavioral defects. Cochlin is the main otolith component and the determinant of the crystalline form of calcium carbonate (Leventea et al., 2021). Xiong et al. (2020) detected cochin- tomoprotein (CTP) in the middle ear, the shortest isoform of cochlin, which is an endolymph-specific protein that is not expressed in blood, cerebrospinal fluid and saliva, but is highly expressed in inner ear lymph fluid and can be used as a diagnostic biochemical marker for endolymphatic fistula.

### Otolith anchoring proteins and otolith functional development

#### Otogelin

Otogelin is a non-collagenous component of the cell-free gel structure covering the sensory epithelium of the inner ear, including the covering membrane (TM) in the cochlea, the membranes of the utricule and saccule, and the cell-free material at the apical part of the semicircular canal jugular in the vestibular organ (Avan et al., 2019). The sensory epithelium of the cochlea (organ of Corti) is covered by a cell-free structure, the TM, which contains two types of sensory cells: inner hair cells and outer hair cells. The outer hair cells are the mechanical actuators of the cochlea and their function consists of anchoring their tallest stereocilia to the covering membrane. The long, immobile motile cilia on the hair cells tether the otolith, and a small number of motile cilia at the poles of the utricle contribute to the accuracy of otolith tethering, but neither the presence nor the movement of the cilia is necessary for this process. Instead, otolith tethering is dependent on the presence of hair cells and the function of Otogelin (Whitfield, 2020). It has been shown that Otogelin also plays an important role in otolith seeding in zebrafish, revealing that Otogelin may play an important anchoring role in otolith formation (Stooke-Vaughan et al., 2015). Otogelin and Otogelin-like are TM proteins associated with secreted epithelial mucins, and defects in either result in DFNB18B and DFNB84B hereditary deafness, both of which are respectively characterized by congenital mild to moderate hearing impairment (Avan et al., 2019). In addition, mutations in the OTOG gene encoding Otogelin cause moderate hearing impairment, which may be associated with vestibular dysfunction in humans (Schraders et al., 2012).

#### α-tectorin and β-tectorin

Both α-tectorin and β-tectorin are non-collagenous glycoprotein components of the cochlear covering membrane (TM). α-tectorin and β-tectorin form a dense matrix within the TM and are used to cross-link type II collagen fibers to adhere the TM to the helical rim (Andrade et al., 2016). In addition, the maintenance of otolith adhesion to sensory maculae was found to be dependent on α-tectorin in zebrafish, and this adhesion process is essential for otolith growth (Stooke-Vaughan et al., 2015). In humans, mutations in the TECTA gene, which encodes alpha-tectorin, cause moderate to severe hearing loss (Asgharzade et al., 2017). Studies have shown that β-tectorin knockouts in zebrafish show balance defect due to disrupted otolith formation and result in severe defects in otolith formation and function in the inner ear, thus suggesting that β-tectorin can affect otolith formation (Asgharzade et al., 2011). However, the underlying molecular mechanisms need to be further investigated. Furthermore, β-tectorin is not only essential for the development of the inner ear, but also normal auditory function. Mutations in its gene can also lead to hearing loss (Zou et al., 2006).

### Other factors that regulate and maintain otolith development

#### Otopetrin-1 and otopetrin-2

Tu et al. (2018) identified Otopetrin1 (Otop1), a proton-selective channel involved in gravity sensing in the vestibular system, as a protein essential for otolith development. It has been shown that Otop1 knockout mice lack functional otoliths and that this leads to vestibular dysfunction (Khan et al., 2019). Past reports have shown that Otop1 is expressed in the extra-striatal region of the mouse saccule and utricle and that Otop1 is enriched in calcium to maintain otolith nucleation, growth, and maintenance (Kim et al., 2010). The otolith is rich in calcium for nuclear formation, growth, and maintenance. During otolith mineralization, Otop1 acts as a receptor for extracellular calcium concentrations near the vestibular support cells, responding to ATP in the endolymph to increase intracellular calcium levels. Its mutation leads to phenotypic imbalance and selective involvement in otolith damage, and Otop1 is likely to function similarly in the human saccule (Lopez et al., 2019). In mouse and human vestibular hair cells, Otop1, Otop2, and Otop3 may function as proton channels (Tu et al., 2018). The differential expression of Otop2 in human saccular-supporting cells suggests an important role in the dynamic balance of vestibular sensory periphery and otolith maintenance, although further studies are needed (Lopez et al., 2019). However, further research is needed. In conclusion, Otop1 is an essential protein for otolith development and maintenance, and Otop2 may play the same role as Otop1 in otolith maintenance (Lopez et al., 2019). Otop2 may play the same role as Otopetrin1 in otolith maintenance.

#### Plasma membrane calcium ATPase isomer 2

Plasma membrane calcium ATPase (PMCA) is a calmodulin-sensitive plasma membrane calcium ATPase. The lack of otolith formation is more likely due to inhibition of Ca^2+^ transport and CaCO_3_ deposition. The PMCA isoform ATP2b1a is an active calcium transporter protein that is essential for normal development and function of the inner ear in zebrafish larvae, and knockout of ATP2b1a in embryos results in larvae exhibiting homeostasis defects, reduced otolith proliferation, ectopic otolith localization and occasional fusion (Han et al., 2019). The PMCA2 pump is expressed at high levels in vestibular and outer hair cells in the static cilia and parietal membrane. It pumps Ca^2+^ out of the cell at a relatively high and constant rate. Inner ear hair cells detect acoustic stimuli, inertia, or gravity through the deflection of their apical stereocilia. The current consensus is that the deflection of the stereocilia bundle toward the higher stereocilia increases the tension in the terminal link and that this mechanical stimulus is transmitted to the cation-selective mechano-transduction (MET) channel located at the top of the stereocilia and opened to allow cation influx into the cell, where Ca^2+^ entering through the MET channel is sequestered by the buffer in the stereocilia and then passed PMCA2w/a pump to and from the endolymph. the PMCA2w/a variant is thought to increase calcium ions near the hair bundle and may be related to the complexity of decidual structures on hair cells in the vestibular system, where the pump also contributes to otolith formation and maintenance (Bortolozzi and Mammano, 2018). PMCA2w/a missense mutations cause deafness and homeostasis disorders in humans (Bortolozzi and Mammano, 2018).

#### NOx (Nicotinamide adenine dinucleotide phosphate oxidases)

Nicotinamide adenine dinucleotide phosphate (NADPH) oxidases (NOx) represent an important family of enzymes that produce reactive oxygen species (ROS) in a regulated manner for host oxidative defense and redox-based signaling (Schiffers et al., 2021). The activity of NOx is regulated by partners such as p22phox, NOx organizers (Noxo1, p47phox, and p40phox), and NOx activators (Noxa1 and p67phox). Noxo1 mRNA is widely expressed in the embryonic inner ear. It has been demonstrated that ROS production by Noxo1-dependent vestibular oxidases is essential for otolith formation, and the absence of the calcium carbonate component in otoliths of Noxo1 mutants suggests that the process of otolithogenesis is impaired (Kiss et al., 2006). Both the inner ear vestibule and cochlea have high levels of NOX3 mRNA expression, and OC90 and NADPH oxidase Nox3, an essential regulatory protein for otolith formation, are functionally interconnected and enhance each other’s functions, which are essential for otolith formation and maintenance (Xu et al., 2021). It is unclear how NOx and its associated proteins mediate otolith formation. The major proteins of otoliths (e.g., OC90) may bind to phospholipids in the vesicle membrane of globules (Ca^2+^-rich vesicles) in an HCO_3_^–^ rich environment and undergo conformational changes triggered by ROS generated by NOx3 located in the vesicle plasma membrane. This promotes the nucleation of calcite crystals from calcium supplied by the vesicles and bicarbonate ions from endolymph. In conclusion, Nox is a major otolith component and essential for calcium carbonate crystal formation. However, NOX-derived ROS are also implicated in the pathophysiology of sensorineural hearing loss. The absence of functional NOX3 enhances the recovery phase of hearing after noise trauma. This opens an interesting clinical window for pharmacological or molecular interventions aimed at preventing noise-induced hearing loss (Rousset et al., 2022).

#### Carbonic anhydrase

Carbonic anhydrase (CA), which catalyzes the hydration of CO_2_ to produce bicarbonate and protons, has been proposed to regulate potassium homeostasis and cochlear potential in the mammalian cochlea. There are 16 known mammalian CA isozymes (Wu et al., 2013). These CA isoforms play a central role in the transport of calcium, protons, and bicarbonate ions, and thus regulates optimal pH regulation and fluid balance in the inner ear (Tohse et al., 2006). Carbonic anhydrase (CA) may also regulate otolith development and maintenance by providing HCO3^–^ and maintaining proper pH. CA is expressed in inner ear hair cells and catalyzes the formation of endolymph from carbonate ions for otolith calcification. Studies have reported that treatment of zebrafish embryos with the carbonic anhydrase inhibitor sulforaphane inhibits otolith formation (Matsumoto et al., 2017). The CA inhibitor ethoxyzolamide inhibits the accumulation of bicarbonate in the endolymph and otolith, inhibits otolith calcification, and induces apoptosis of inner ear hair cells. Apoptosis of hair cells can lead to varying degrees of hearing loss (Tohse and Mugiya, 2001; Liu et al., 2022).

## Discussion

This review summarizes the inner ear proteins associated with the development and maintenance of otolith and describes the progress in the study of otolith-related proteins and their association with vestibular-related diseases. Little is known about the molecular processes involved in otolith maintenance and human vestibular pathology. Slow and progressive otolith degeneration is part of the normal aging process and can lead to severe balance disorders resulting in falls in the elderly (Agrawal et al., 2012). Otolith degeneration can be caused by ototoxic drugs, infection, trauma and aging. To date, several otolith-related proteins have been investigated and these otolith-related proteins have been studied at the cellular and subcellular levels to show that otoliths begin to degenerate in the fifth decade (Ross et al., 1976). Therefore, determining the protein composition of otoliths in the human saccule and utricle and studying their changes with age and disease can help to clarify the etiology of vestibular pathologies (Lopez et al., 2019). In this review, aggregated data present a clearer picture of otolith-associated proteins in vestibular disorders.

### Otolithic proteins associated with benign paroxysmal positional vertigo

Benign paroxysmal positional vertigo is one of the most common otoconia-related balance disorders, and there are no laboratory indicators available to determine the diagnosis of BPPV (Wu et al., 2020). In contrast, recent studies have found that Otoconin-90 can be quantitatively detected in peripheral blood and suggest that Otoconin-90 can be used as a biomarker for BPPV and has clinical significance for the diagnosis of certain patients with BPPV, such as elderly or posttraumatic patients who have difficulty performing diagnostic postural maneuvers, or patients who had nystagmu or satypical symptoms (Bi et al., 2021). In addition, serum otolin-1 is quantifiable in BPPV patients, with elevated levels of Otolin-1 in BPPV patients, who are sensitive to BPPV (Yadav et al., 2021), high serum otolin-1 levels associated with increased risk of BPPV recurrence (Fan et al., 2022). A case-control study found that serum levels of otolin-1 were significantly higher in patients with BPPV than in patients with non-BPPV (IIrugu et al., 2021). Furthermore, high levels of serum Otolin-1 (>299.45 pg/ml) can be used as a biomarker to differentiate patients with BPPV from healthy controls, suggesting that serum Otolin-1 could be used as a biomarker for BPPV episodes and could be used clinically to promote better management of BPPV (Wu et al., 2020). From a clinical perspective, biomarkers of otolith degeneration can help as a potential diagnostic modality, therapeutic target, and predictive, and therapeutic response monitoring (Yadav et al., 2021).

### Otolithic proteins associated with Meniere’s disease and hearing loss

Human missense mutations in Cochlin are related to autosomal dominant hearing loss (DFNA9) (Robertson et al., 1998; Py et al., 2013). Consistent with Cochlin determining CaCO_3_ crystallization, Cochlin mutations in both human and mouse cause vestibular malfunction (Khetarpal et al., 1991; Jones et al., 2011), while some human mutations also result in vertigo (Khetarpal et al., 1991). Notably, most of the mutations in Cochlin are dominant missense mutations in the N-terminal factor C homolog and result in a form of Cochlin with deposition in the vestibular and cochlear labyrinths (Robertson et al., 1998). There are data suggesting that Cochlin is overexpressed in the vestibular endorgans of subjects with persistent Ménière’s disease (Calzada et al., 2012) and in autoimmune inner ear disease (Pathak et al., 2013), which may contribute to the dysregulation of the environmental balance within the inner ear in Ménière’s disease (Calzada et al., 2012). While α-tectorin is involved in the pathophysiology of Ménière’s disease, the shift code deletion of the TECTA gene may be involved in TM formation through altered GPI-anchored signaling leading to an altered clinical phenotype (Roman-Naranjo et al., 2022). Mutations in the human TECTA gene encoding alpha-tectorin cause hearing loss (Asgharzade et al., 2017). Missense mutations in PMCA2w/a cause deafness and balance disorders in humans (Bortolozzi and Mammano, 2018). Mutations in the OTOG gene encoding Otogelin in humans result in moderate hearing impairment (Schraders et al., 2012). Interestingly, one study found that 15 of 46 Ménière’s disease families (33%) showed at least one rare missense variant in the OTOG gene, a finding that supports OTOG as an associated gene in familial Ménière’s disease and lays the foundation for genetic testing for Ménière’s disease (Roman-Naranjo et al., 2020). These findings provide new ideas for genetic testing in patients with comorbid hearing loss in vestibular disease, allowing for the detection of the Cochlin, OTOG, PMCA2, and TECTA genes. If these disorders are detected in a timely manner, early intervention in balance disorders or reduction in the degree of hearing loss can be achieved.

Understanding the function of otolith-associated proteins may provide new ideas for the prevention and diagnosis of vestibular disease and provide a basis for otolith-associated proteins as potential biomarkers of vestibular disease and for conducting corresponding genetic testing. Nevertheless, further research is needed to determine the role of many of the newly identified proteins (Parham and Dyhrfjeld-Johnsen, 2016). Nevertheless, there are indeed certain limitations to our studies, one of the fixed limitations of search-based studies being that the search terms limit them. In this particular area, not all researchers mentioned the term “otolith-related protein” in the keywords, study titles, or conclusions of their publications.

In the future, it is hoped that more otolith-related proteins will be studied and newer diagnostic or therapeutic ideas will be brought to clinic. It is important to be able to determine the use and utility of each otolithic protein in specific vestibular disorders, as well as to combine functional biomarkers and include evoked potentials of hearing and balance-related tests such as vestibular evoked myogenic potentials (VEMPs), posturography, and rotational chair responses which may provide new insights into the diagnosis, treatment or prognosis of vestibular diseases including Meniere’s disease and BPPV. Revisiting the otolith-associated proteins, focusing on the most clinically applicable studies to fill in the knowledge gaps, and linking them to vestibular disorders related diseases, and exploring possible biomarkers and genetic tests, may be the future direction of this research.

## Author contributions

SH: data curation and writing – original draft, review and editing. SQ: supervision and funding acquisition. Both authors contributed to the article and approved the submitted version.

## Conflict of interest

The authors declare that the research was conducted in the absence of any commercial or financial relationships that could be construed as a potential conflict of interest.

## Publisher’s note

All claims expressed in this article are solely those of the authors and do not necessarily represent those of their affiliated organizations, or those of the publisher, the editors and the reviewers. Any product that may be evaluated in this article, or claim that may be made by its manufacturer, is not guaranteed or endorsed by the publisher.
